# Development and clinical evaluation of a simple optical method to detect and measure patient external motion

**DOI:** 10.1120/jacmp.v16i5.5524

**Published:** 2015-09-08

**Authors:** Benigno Barbés, Juan Diego Azcona, Elena Prieto, José Manuel de Foronda, Marina García, Javier Burguete

**Affiliations:** ^1^ Servicio de Radiofísica Clínica Universidad de Navarra Pamplona Spain; ^2^ IdiSNA (Instituto de Investigación Sanitaria de Navarra) Recinto de Complejo Hospitalario de Navarra c/Irunlarrea Pamplona Spain; ^3^ Tecnun Universidad de Navarra San Sebastián Spain; ^4^ Departamento de Física y Matemática Aplicada Facultad de Ciencias, Universidad de Navarra Pamplona Spain

**Keywords:** radiotherapy, motion, optical, gating

## Abstract

A simple and independent system to detect and measure the position of a number of points in space was devised and implemented. Its application aimed to detect patient motion during radiotherapy treatments, alert of out‐of‐tolerances motion, and record the trajectories for subsequent studies. The system obtains the 3D position of points in space, through its projections in 2D images recorded by two cameras. It tracks black dots on a white sticker placed on the surface of the moving object. The system was tested with linear displacements of a phantom, circular trajectories of a rotating disk, oscillations of an in‐house phantom, and oscillations of a 4D phantom. It was also used to track 461 trajectories of points on the surface of patients during their radiotherapy treatments. Trajectories of several points were reproduced with accuracy better than 0.3 mm in the three spatial directions. The system was able to follow periodic motion with amplitudes lower than 0.5 mm, to follow trajectories of rotating points at speeds up to 11.5 cm/s, and to track accurately the motion of a respiratory phantom. The technique has been used to track the motion of patients during radiotherapy and to analyze that motion. The method is flexible. Its installation and calibration are simple and quick. It is easy to use and can be implemented at a very affordable price. Data collection does not involve any discomfort to the patient and does not delay the treatment, so the system can be used routinely in all treatments. It has an accuracy similar to that of other, more sophisticated, commercially available systems. It is suitable to implement a gating system or any other application requiring motion detection, such as 4D CT, MRI or PET.

PACS numbers: 87.55.N, 87.56.Da

## I. INTRODUCTION

The International Commission on Radiation Units recommends that the accuracy in dose delivery should be within 5%.^1^, ^2^ As stated in the introduction of IAEA TRS 398, “Modern diagnostic tools for the determination of the target volume, 3D commercial treatment planning systems and advanced accelerators for irradiation, can only be fully utilized if there is high accuracy in dose determination and delivery.”^(3)^ Radiobiological and clinical studies suggest that a dose reduction of 7% to 15% to a portion of the tumor can significantly reduce the local tumor control probability (TCP).^4^, ^5^ Patient motion during a radiotherapy session is a major source of uncertainty in these therapies,^1^, ^6^ especially in complex treatments.^(7)^ Dealing with potential motion involves three issues: to limit motion, to handle the residual motion (shorten treatment time, increase safety margins to the organs, or other complex techniques as beam gating and tumor tracking), and to estimate the consequences of residual motion.^6^, ^8^ For these three issues, the extent of the motion must be known.

Among all kinds of motion, that due to respiration is the largest encountered in practice, and several approaches have been made to reduce its influence.^9^, ^10^, ^11^, ^12^, ^13^, ^14^, ^15^, ^16^ However, there are many other types of motion, some of them leading to significant problems. For example, variable filling of the rectum and bladder,^17^, ^18^ peristalsis, and cardiac motion can all cause difficulties;^(6)^ and involuntary changes in patient position over the couch caused by anxiety, discomfort or other causes, are also frequent.

Several stereotactic radiosurgery (SRS) methods are based on rigid frames attached to the skull to fix it, both using screws or a vacuum bite block.^(19)^ Other systems replace them with less precise immobilization devices such as thermoplastic mask, that has been reported to allow head motion of more than 2–3 mm.^20^, ^21^ Due to this limitation, it is necessary to perform head tracking to check if such drifts exceed SRS tolerances, and correct them. Different tracking systems have been developed to evaluate these drifts.^22^, ^23^, ^24^, ^25^

Most of the patient positioning verification systems, like planar images or cone‐beam computed tomography (CBCT) with MV electronic portal imaging device (EPID) or kV on‐board imaging (OBI) system, use ionizing radiation to obtain an image of the patient; so they impart an additional dose of radiation to patients.^26^, ^27^, ^28^ Other noninvasive methods track the external contour of the patient: optical infrared (IR) tracking of external markers,^16^, ^23^, ^25^ optical surface image of patient,^22^, ^29^, ^30^, ^31^, ^32^, ^33^ time‐of‐flight sensors,^(34)^ or tracking of external objects on the surface of the patient.^(12)^ External tracking methods have the drawback of using surrogates of the actual organ motion, so it is necessary to verify the reproducibility between external and internal motion. Several authors^35^, ^36^ have found good correlation between external marks and internal motion, also depending on the kind of breathing,^(36)^ especially when using multiple markers or surface monitoring,^34^, ^37^, ^38^, ^39^, ^40^ at least for tumors in the lung, liver, pancreas, and other thoracic and upper abdominal structures.^(41)^

In this work a novel method of motion tracking, with high accuracy, speed, and sensitivity, was devised and tested. It was also applied to track, record, and analyze trajectories of several independent points on the surface of the patients during radiotherapy treatments. The system is simple and can be installed in any treatment room at a very affordable price; it is very easy to use and does not need training; it does not introduce discomfort to the patient (only requires to stick a label on the skin), nor delay of the treatment; and it is completely harmless, so it can be used routinely in every treatment session.

## II. MATERIALS AND METHODS

### A. Algorithm

Several techniques have been proposed for vision metrology, based on the original work of Tsai.^(42)^ In this work we developed an adapted version using basic geometry, suitable to our needs. A point (x,y,z) in the space is projected in the position ($u_{1},v_{1}$) for images of camera 1, and ($u_{2},v_{2}$) for images of camera 2. A pair of functions, $f_{1},f_{2}$, are required satisfying the property:

(1) $$\left(u_{1},v_{1})=f_{1}(x,y,z)\;and\;(u_{2},v_{2})=f_{2}(x,y,z\right)$$

In addition, functions $f_{1},f_{2}$ have to be bijective in order to make it possible to obtain the position of a point in the 3D space from its position in the two 2D images:

(2) $$\left(x,y,z)=f^{-1}(u_{1},v_{1},u_{2},v_{2}\right)$$

In absence of aberration in the optical systems, the transformation from (x,y,z) coordinates in the space to each one of the coordinates ($u_{1},v_{1}$), ($u_{2},v_{2}$) in one of the image projections, is the composition of a linear deformation L with a perspective projection P. It can be expressed as:

(3) $$\left(\begin{array}{l} u_{i}\\ v_{i} \end{array}\right)=\frac{1}{w_{i}}L_{i}\left(\begin{array}{l} x\\ y\\ z\\ 1 \end{array}\right)\;\;\text{being}\;\;L_{i}=\left(\begin{array}{llll} \alpha_{i} & \beta_{i} & \gamma_{i} & \delta_{i}\\ \varepsilon_{i} & \zeta_{i} & \eta_{i} & \theta_{i} \end{array}\right)\;\;i=1,2$$

with

(4) $$w_{i}=P_{i}\left(\begin{array}{l} x\\ y\\ z\\ 1 \end{array}\right)\;\text{being}\;P_{i}=(\begin{array}{llll} \iota_{i} & \kappa_{i} & \lambda_{i} & \mu_{i} \end{array})\;i=1,2$$

So, 12 coefficients for each transformation are needed (24 in total). From each pair of measurements ($u_{1},v_{1}$) ($u_{2},v_{2}$), corresponding to a point (x,y,z), we have two equations; so six calibration points are necessary to obtain the 12 coefficients of each transformation.

Developing on (3), (4), we obtain a system of two equations for each one of the two cameras (subscript i), and for each one of the six point (superscript j):

(5) $$\left(\begin{array}{lll} \alpha_{i}-u_{i}^{j}\iota_{i} & \beta_{i}-u_{i}^{j}k_{i} & \gamma_{i}-u_{i}^{j}\lambda_{i}\\ \varepsilon_{i}-u_{i}^{j}\iota_{i} & \zeta_{i}-u_{i}^{j}\kappa_{i} & \eta_{i}-u_{i}^{j}\kappa_{i} \end{array}\right)\left(\begin{array}{l} x^{i}\\ y^{i}\\ z^{i} \end{array}\right)=\left(\begin{array}{l} -\delta_{i}+u_{i}^{j}\mu_{i}\\ -\theta_{i}+v_{i}^{j}\mu_{i} \end{array}\right)$$

where $\alpha_{i}...\mu_{i}$ are the 12 coefficients needed for each projection $f_{i}$ of Eq. (1). Solving this set of 12 equations for each camera, we determine $L_{i}$ and $P_{i}$.

Once these matrices are obtained, it is possible to determine the position of a point (x,y,z) from the projections in the cameras ($u_{1},v_{1}$) and ($u_{2},v_{2}$), solving the system of equations:

(6) $$\left(\begin{array}{lll} \alpha_{1}-u_{1}\iota_{1} & \beta_{1}-u_{1}\kappa_{1} & \gamma_{1}-u_{1}\lambda_{1}\\ \varepsilon_{1}-v_{1}\iota_{1} & \zeta_{1}-v_{1}\kappa_{1} & \eta_{1}-v_{1}\lambda_{1}\\ \alpha_{2}-u_{2}\iota_{2} & \beta_{2}-u_{2}\kappa_{2} & \gamma_{2}-u_{2}\lambda_{2}\\ \varepsilon_{2}-v_{2}\iota_{2} & \zeta_{2}-v_{2}\kappa_{2} & \eta_{2}-v_{2}\lambda_{2} \end{array}\right)\left(\begin{array}{l} x\\ y\\ z \end{array}\right)=\left(\begin{array}{l} -\delta_{1}+u_{1}\mu_{1}\\ -\theta_{1}+v_{1}\mu_{1}\\ -\delta_{2}+u_{2}\mu_{2}\\ -\theta_{2}+v_{2}\mu_{2} \end{array}\right)$$

The system of equations is over‐dimensioned, consisting of four equations and three unknowns, which leads to four solutions (x,y,z). Using two cameras placed at the same height, two of these solutions always have a greater uncertainty because they correspond to intersections between almost parallel planes. These solutions are disregarded, and only the pair of low uncertainty solutions is taken. On the other hand, minimal changes in cameras orientation or in video temporal synchronization produce discrepancies between the two solutions used for the procedure, so the system can detect these problems and alert the user, who will have to perform a recalibration (2 min).

### B. Equipment

**Figure 1:**
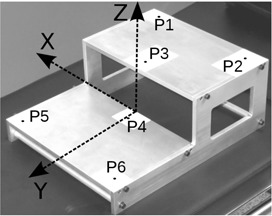
In‐house calibration phantom showing the six accurately located calibration points.

The recording system consists of two cameras and a digital video recorder X–Motion Premium 4 (Presntco, Barcelona, Spain). We have fitted two sets of cameras in two scenarios: the bunker of a radiotherapy linear accelerator (linac) (two Intellisense CC 2330P cameras, 752×582 px; Honeywell, Morristown, NJ), and the room of a CT scanner (two MediaWave Varifocal 27X, 1/4ʺ CC, 752×582 px, Qualiano, Italy). For the calibration of the imaging system, an aluminum structure was manufactured, and black dots (4 mm diameter) printed on the center of six 65×45 mm white stickers were located on it with their positions known with accuracy of ±0.5 mm (see Fig. 1).

**Figure 2:**
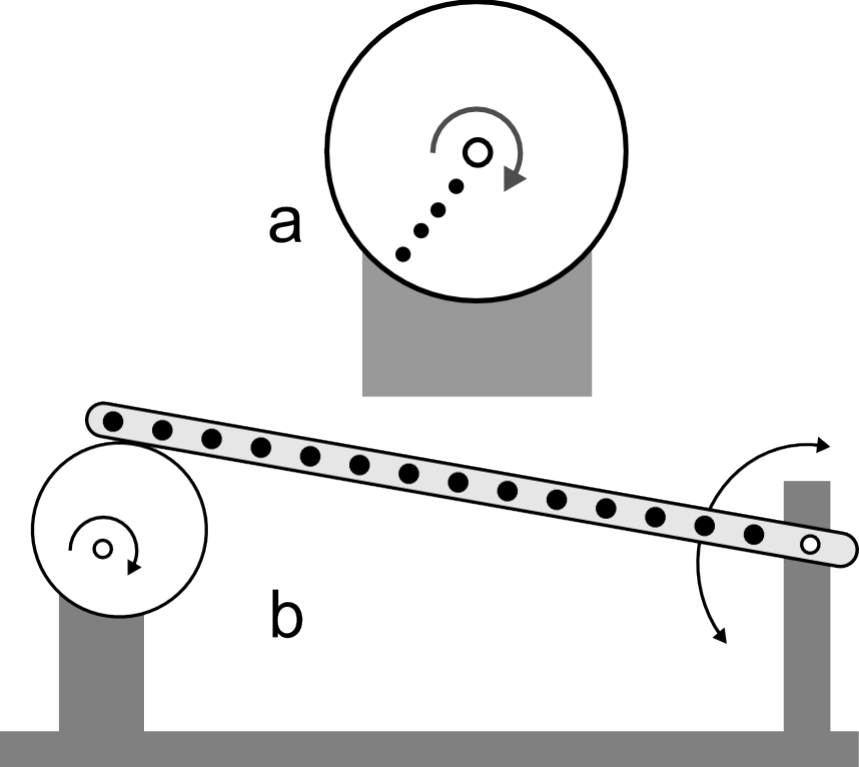
Sketch of (a) the in‐house phantom made to perform circular motion and (b) the in‐house phantom made to perform oscillations of low amplitude. As the eccentric disk rotates, the points of the bar move up and down.

To measure large displacements of a point, the linac couch (Siemens ZXT; Siemens AG, Munich, Germany) display was used, precision of ±1 mm in all three directions. To measure small displacements, a structure moved by micrometer screws (Parker Hannifin Daedal, Irwin, PA) was used, allowing ±0.03 mm precision. Circular trajectories were performed using an in‐house phantom; a motor rotates a disk around its axis, at 1 rpm speed (see Fig. 2(a)). To test the tracking of respiratory motion, a respiratory phantom, manufactured by Anzai Medical Co. (Tokyo, Japan) for the Anzai AZ–733V respiratory gating system, was used. It describes a single direction oscillatory motion of 2 cm of amplitude, with 10 rpm and 15 rpm frequencies, and two kinds of motion — respiration cycle emulation or sinusoid. Note that Anzai phantom motion (±1 mm) is not precise enough to check spatial accuracy of our system.

A second in‐house phantom was made to simulate sinusoid motions of lower amplitude. A diagram is shown in Fig. 2(b). An electric motor rotates a disk around an eccentric axis. One extreme of a rigid bar is supported on the disk and the other extreme can rotate around a fixed axis. Fourteen dots were printed on the bar, at different distances from the axis. The maximum angle of the oscillating bar is 0.7°, so the trajectories of the points can be considered straight, with less than 0.003% error (difference between the arc and its sine). The amplitudes of the displacements are between 0.46±0.02 mm and 8.4±0.1 mm.

The videos were recorded with a resolution of 704×576 pixels, 25 frames per second (fps), and AVI format (ITU H.264 codec). Subsequently they were decomposed into frames (25 images per second). The program that analyzes the motion using the images was implemented in MATLAB (MathWorks, ver. 2008b; Natick, MA). An algorithm to fit data to a sine function was implemented in Python.

For real‐time points tracking, a frame grabber card DGF/MC4/PCIe from The Imaging Source (Bremen, Germany) was used. It allows up to 704×576 pixels images at 25 fps. The card was controlled by a HP Z420 workstation (Intel Xeon E5‐1620 3.6GHz, 8GB RAM; Intel Corporation, Santa Clara, CA).

Illumination conditions were measured with a light meter HIBOK 30 (Tecno y Medida S.L., Barcelona, Spain).

### C. System implementation

The calibration phantom of Fig. 1 has six marks, at positions known with high accuracy, all of them in a 100×350×250 mm volume. The in‐house MATLAB program uses a pair of images of the phantom, one per each camera. On these two images, the user indicates the approximate location of the six calibration points. A localization algorithm in two steps obtains the exact two‐dimensional coordinates of the six points in the 2D coordinates system of the images, as the centroid of the detected spots, with precision better than a pixel. The three‐dimensional coordinates of the six points in the real space have been introduced in the program. With these data, the script calculates the 24 coefficients of Eq. (5) and is able to solve Eq. (6), obtaining the real coordinates (x,y,z) through the projections on the cameras ($u_{1},v_{1}$) ($u_{2},v_{2}$). It is worth noting that the system provides the absolute position of a point in space, relative to the coordinates in which the phantom calibration points were defined (arbitrary origin). However, for all measurements done in this work, only relative displacements of points have been measured, not their absolute positions in space. The calibration remains valid as long as positions and angles of the cameras do not change.

In order to obtain an accurate 3D position of the point, it is necessary to know the positions on the images with subpixel accuracy. To do it, the program starts with a first approximation of the positions of the dots, finds the maxima pixel values around these positions, and selects a region around the maxima. Then, it interpolates the pixel values inside that region, and obtains the centroid of the interpolated region, with subpixel precision. A possible drawback is that our approach uses a circular dot placed over the surface of the patient that can be deformed by the optical system (becoming an ellipse) or by the surface curvature (becoming a bean shaped spot). The system can account for the first deformation. To avoid the second, stickers are placed in anatomical positions where the deformation is not too big.

**Figure 3:**
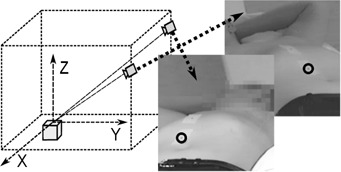
Sketch of the measurement setup, and an example of the paired images obtained. The circles mark the points that have to be tracked.

The cameras were attached to the bunker walls using a support system. They were located at a distance of about 4 m from each other, at a horizontal distance of about 4 m from the isocenter of the linac, and at a height of about 1.5 m from the isocenter (see Fig. 3). The position of the cameras is not relevant. If there is any inaccessible point, cameras can be easily placed in other positions; in that case, the system has to be recalibrated. To the best of our knowledge, most of the commercial optical tracking systems do not allow the user to change the position of the cameras in a short time. On the other hand, the system is very sensitive to small accidental changes on the position and angle of the cameras between calibration and measure, but these changes can be easily detected, because they produce discrepancies among the solutions of Eq. (6). One mark was placed on the wall of the bunker inside the field of view of the cameras. Checking if these marks are in the same position on calibration and measurement images, it is possible to test if there was any motion in the cameras, and if this occurs, the system calibration must be repeated.

An in‐house program was developed to study the motion of one or more points. The workflow is as follows. An adhesive label (minimum size 20×30 mm), with a 4 mm diameter black spot printed on it, is placed on each zone to track (see Fig. 3). Two cameras record the points moving over time, obtaining a set of paired images. In the first pair of images, the user indicates a first approximation of the position of the points, and the program calculates the precise positions using the method described before. In the subsequent pairs of images, the software uses the last position of the points as an approximate location. From the (2D) positions of the points in each frame, the real positions (3D) are calculated, so the 3D trajectories of the marks are obtained independently. Temporal synchronization of the pairs of images was tested. Barrel distortion was also evaluated and correction was done for Intellisense cameras; it was unnecessary for the MediaWave ones. No further corrections were necessary to obtain enough accuracy.

**Figure 4:**
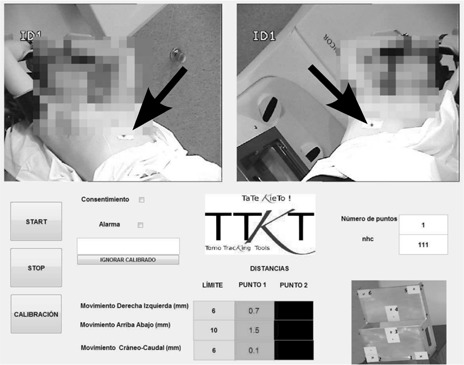
A view of the user interface of the program. The algorithm is tracking the motion of only one point placed on the patient skin (black arrow). Motion magnitudes in mm are shown besides their respective tolerances; they are depicted over green color if tolerances are being met, or over yellow or red colors otherwise.

A second in‐house application to detect, track, and record motion of the patients, was implemented (Fig. 4). Real‐time images were obtained using a frame grabber card. As signals are not multiplexed, the pairs of images grabbed are synchronized; from them, 3D positions are calculated as in the former program. The application shows the displacements of selected points in three directions, with the patient lying on the couch: X axis from left to right, Y axis postero–anterior, and Z craniocaudal. It also alerts if out‐of‐tolerance motion is detected, at which instance the display turns red, and an alarm sounds. If we have the patient's consent, the program can store the trajectories of the tracked points in an anonymized file. Whenever the algorithm detects a big discrepancy between the two solutions of Eq. (6), it asks the user to perform a new calibration, to avoid erroneous results.

### D. Measurement setup

#### D.1 Measurements accuracy

The upper bound for the displacements of the points on the surface of an immobilized patient is lower than some centimeters (taking into account the breathing cycle) and these movements can be measured with our system, as it will be shown in the Materials & Methods section D.2. But we wanted to know the lower bound of our system, so we characterized the accuracy with which we can determine the displacement of an arbitrary set of points. To test it, 16 points were placed on a structure that can be moved with micrometer screws. Taking the initial position of each point as origin, seven displacements of the whole structure (from 1.27 to 10.16±0.03 mm) were done in X direction, six (from 1.27 to 8.89±0.03 mm) in Y direction, and six (from 1.27 to 8.89±0.03 mm) in Z direction.

#### D.2 Motion tracking

To validate the precision of the algorithm for large displacements, the linac couch was used to perform linear motion. A digital display showed its position with ±1 mm precision. Several linear trajectories were described by the couch, and the motion of a mark on its surface was tracked by the system. Maximum lengths of the trajectories were 5 cm in x‐axis, 20 cm in y‐axis, and 10 cm in z‐axis.

Circular motion was performed using the in‐house phantom (Fig. 2(a)) with the disk rotating parallel to planes XY and XZ. Several points to track were depicted at different distances from the center of the disk.

In order to test the system in oscillatory motion, the respiratory simulation Anzai phantom was placed so that its motion followed several straight trajectories — each one of the x‐, y‐, and z‐axes. The amplitude of the oscillation was always 20±1 mm, the frequencies were 10 and 15 rpm, and motion was both sinusoid and quasi‐respiratory curve.

To test the ability of the system to track more precise and lower amplitude oscillatory motion, the in‐house phantom of Fig. 2(b) was used. It was placed on XY plane, oscillating in Z direction.

#### D.3 Study with patients

**Figure 5:**
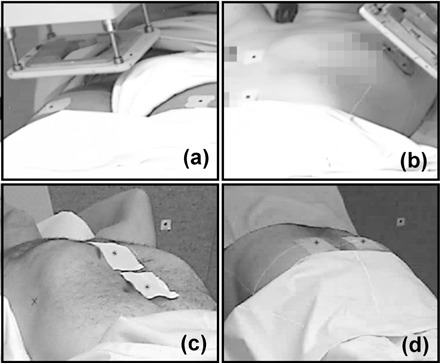
Examples of the position of the labels on patients surface: (a) bladder with electrons; (b) breast with electron beam applicator; (c) lung; (d) rectum.

The system was tested with 23 volunteer patients (21 of breast tumors, 1 of lung tumor and 1 of bladder tumor), each of them being treated between 4 and 23 sessions. Between one and three labels, with a 4 mm diameter black spot, were placed over the surface of the patients during the therapy, and were tracked by the system. A total of 461 point trajectories were obtained and analyzed. Figure 5 shows four examples of labels positions.

In this work, only the studies of 21 breast tumor patients will be shown (399 trajectories), in order to analyze trajectories with similar characteristics. All breast patients received similar treatments, and were positioned using the C–Qual breastboard manufactured by CIVCO Medical Solutions (Kalona, IA). We present preliminary data for the analysis of two different kinds of motion of the patients: breathing motion and nonbreathing motion (due to random or accommodation displacements). Each data series include both kinds of motion. To separate them, we applied a smoothing algorithm. First, a Fast Fourier Transform (FFT) was computed for each series, to obtain the average breathing period. Then, a moving average, using a window twice the breathing period, was used to split global motion into breathing and nonbreathing displacements.

#### D.4 Respiration tracking

Once the feasibility of the system to record the respiratory motion was demonstrated, we extended the system to track surface and volumetric features of thoracic and abdominal motion. Fifteen black points were printed on the front side of a white T‐shirt, with 5 cm of distance among them. A healthy volunteer wore the tight fitting T‐shirt in such a way that the T‐shirt cannot slide over the skin, and the system was used to track the respiratory cycle.

#### D.5 Influence of illumination

Different room illuminations were tested, and illuminance on the surface of an object was measured with the light meter before using the system to track the motion of a point on the surface of the object.

## III. RESULTS

### A. Measurements accuracy

Motion described in the Materials & Methods section D.1 was performed, displacements were measured with the novel system, and differences between real and measured positions were computed. Mean deviation (ē) and standard deviation (s) of shifts in mm were ${ē}_{x}=0.01,s_{x}=0.1,{ē}_{y}=0.05,s_{y}=0.1,{ē}_{z}=0.03,s_{z}=0.2$.

### B. Motion tracking

Several displacements of a point were done using the couch of the linac, up to 5 cm in x‐axis, 20 cm in y‐axis, and 10 cm in z‐axis. Measurements of the optical system always agreed with the couch display measurements, within the couch precision (±1 mm).

The system was able to detect and track, with high accuracy, the trajectories of points on the surface of a disk in circular motion of frequency 1 Hz, and radii 3.6 mm, 11.6 mm, and 18.5 mm. In the plane of the motion, the graphic of displacements versus time fits well to sine functions, with $R^{2}$ between 0.9993 and 0.99998, and root mean squared error (RMSE) between 0.03 mm and 0.14 mm. Levenberg–Marquardt algorithm was used, and the definition:

(7) $$RMSE=\sqrt{\sum_{i=1}^{n}\frac{{\left(y_{i}-{\hat{y}}_{i}\right)}^{2}}{n-m}}$$

where *y_i_* are the calculated values, *ŷ_i_* the fitted ones, *n* the number of points, and *m* the number of fitting constants.

It is worth noting that linear speed of the third point was about 11.6 cm/s. The system was not able to track points located further from the center of the disk, but this could be avoided by using cameras with a higher frame rate.

**Figure 6:**
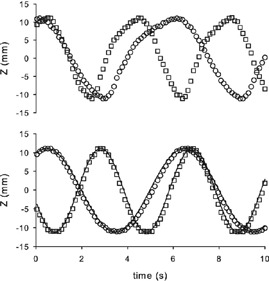
Amplitude of the motion of Anzai phantom in z‐ (vertical) axis, for frequencies 10 rpm (○) and 15 rpm (□), and for quasi‐respiratory (up) and sinusoidal (down) modes. Only one of each three points is represented. Lines are the fitting of the data.

The system tracked the motion of a mark stuck to the mobile part of the Anzai phantom. The six sinusoidal trajectories (two frequencies and three positions on space) could be fitted to sine curves with $R^{2}$ between 0.996 and 0.999, and RMSE between 0.26 mm and 0.52 mm. Figure 6 shows some of the results for both quasi‐respiratory and sinusoidal modes.

The system also correctly tracked the fourteen points of the in‐house phantom sketched in Fig. 2. Motion amplitudes were lower than those of Anzai phantom. Trajectories were fitted to sine curves, being the motion amplitudes one of the fitting parameters. Values of $R^{2}$ were >0.97 for motion with amplitude between 1.2 mm and 8.4 mm. Motion with amplitudes from 0.4 to 1.2 had a $R^{2}>0.74$. Fitted amplitudes always agreed with theoretical ones within ±0.2 mm. Table 1 summarizes the results.

**Table 1:** Summary of the main features of the tracking system, as were obtained using home‐made phantoms

precision for relative motion	<0.3 mm
precision in absolute position for big displacements (20 cm)	<1 mm
maximum speed detected	11.5 cm/s
minimum amplitude of periodic motion detection	<0.4 mm
precision when tracking Anzai phantom	$R^{2}>0.996$
number of independent points tracked	arbitrary^a^

a Only limited by the speed of the computer; up to 20 in the proposed setup.

### C. Patient study

Once the system accuracy was determined, we tested the feasibility of the system to detect and track motion of the patients. We tracked and obtained the 3D trajectories of 461 points on 23 volunteer patients, between one and three points on each patient. In all treatments it was possible to place labels that could be recorded by the cameras. A clear advantage of the system is that it can be employed even when using electron applicators (see Fig. 5).

**Figure 7:**
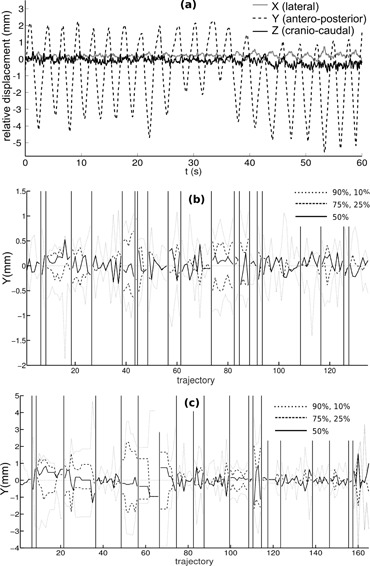
Relative displacements (a), with respect to the initial position, of a point on the thorax of a breast cancer patient, showing respiratory motion in 3D (X, Y, and Z directions); percentiles ((b) and (c)) of respiratory amplitude in antero–posterior (Y) direction in each treatment session, for 21 breast tumor volunteer patients. For patients in (b), points to track were placed on the sternum; for patients in (c), points were on the epigastrium. Vertical lines divide trajectories of different patients.

The system was able to track the respiratory motion of all patients, also in cases of very shallow breathing, and in the three directions of the space; Fig. 7(a) shows an example. In an extreme motion case, the system detected large diaphragmatic motion (20–30 mm in antero–posterior direction) on a patient's lung and adrenal therapy, which suggested further adjustment of the treatment.

To analyze breathing kind motion, we calculated the oscillatory part of the 399 trajectories of the 21 breast tumor patients, and a comparative study was carried out. For the resulting pure respiratory data, we calculated 10, 25, 50, 75, and 90 percentiles. Figures 7(b) and(c) display the results in antero–posterior direction. A positive median means that the patients stay longer in inspiration than in expiration. Differences between thoracic and abdominal breathing can be seen. There are also differences among patients; one of them shows breathing displacements much bigger than those of the other seven.

**Figure 8:**
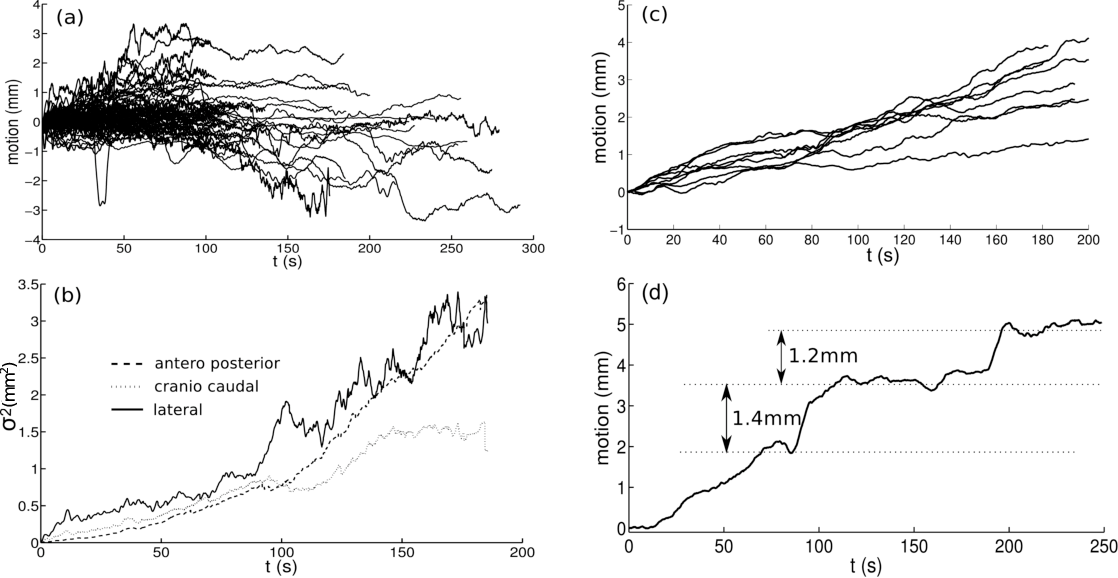
Smoothed trajectories of all points (399) (a) tracked for breast cancer patients, for lateral motion; variance of the 399 motion measurements (b) at each time, for the three axis; all the smoothed trajectories (c), in lateral direction, of a particular patient; a particular smoothed trajectory (d), in left to right direction.

Concerning nonbreathing motion, we subtracted the periodic part of the 399 trajectories, and analyzed the remaining part. As an example, right to left trajectories are depicted in Fig. 8(a). If these movements were purely stochastic, we would expect a diffusive behavior (i.e., a variance ($\sigma^{2}$) that increased linearly with time (Brownian motion)). This was what we found in sternum motion for short times (5 min) (see Fig. 8(b)), especially in antero–posterior and craniocaudal motion. With that tendency, in 2/3 of the patients' sternum zone separate less than 1 mm from its initial position, and 1/3 of them will shift between 1 and 2 mm. Note that these drifts cannot be due to an error in the acquisition system because its accuracy remains below 0.3 mm for long acquisition times.

The global behavior for all trajectories demonstrated that, in average, patients remain at the same position; average position is nearly zero even for large periods (5 min). See for example Fig. 8 (a), in left–right direction. But when each patient was considered separately, clear differences among dataset were found. Figure 8(c) shows an example in which a clear bias to the right can be seen — the patient moved, on average, 3 mm in 5 minutes. Finally, in Fig. 8(d) we present a case when our procedure allowed us to find motion of a patient by steps; it likely was an accommodation motion. Results are compatible with those of more complete studies.^(43)^

It is worth noting that the ability of the system to track motion of independent points on the surface of the patients allows to detect deformations of the surface of the patient — for example, motion of the breast in breast tumor patients.

### D. Respiration tracking

**Figure 9:**
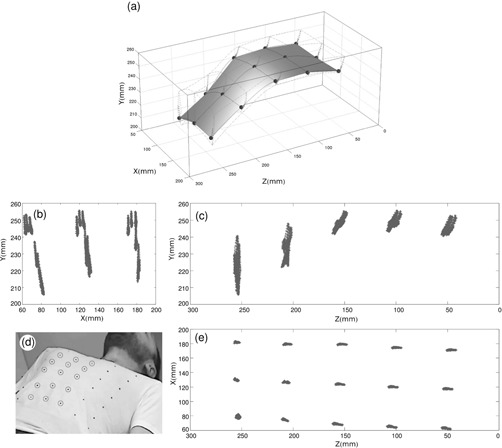
Graph of the trajectories of the 15 central points (a) (marked in the photo) detected by the system; projections ((b),(c),(e)) of the points detected in three planes; photograph (d) of a volunteer wearing the T‐shirt with the marks.

Figure 9 shows how the system tracked 15 points on the surface of the healthy volunteer, and the respiratory motion was correctly detected. For example, it can be seen that points placed on epigastrium zone described almost vertical trajectories, but points on the sternum region moved also in craniocaudal direction.

### E. Room illumination influence

Several points on the surface of an object were tracked by the system under different room illuminations — changing from 10 to 250 lux, or generating moving shadows to switch between 20 and 140 lux. The system was able to accurately track every point in all cases.

## IV. DISCUSSION

A novel method of locating and tracking of points in space has been developed and tested. Although the method could be used in many other fields, the scope of this work was focused in patient tracking for radiotherapy applications. To track the motion of one or more areas on patient surface, the user only has to stick labels on the area(s) and start the program. It does not increase treatment duration nor patient discomfort. It can track the motion (translation and rotation) of head, thorax, bone joints, and any other part of the body. It collects 4D data for each point, with no limitation to the number of them. Calibration is an easy and quick task — about 1 minute, and has only to be repeated if the cameras were moved. All the algorithms and software needed to control the system were developed by the authors.

The method has been proven to be reliable and accurate. It could work well in several circumstances of room illumination. It was able to measure big displacements on three space directions up to 20 cm, with precision better than ±1 mm, and small displacements with precision better than ±0.3 mm. It could also track accurately oscillatory motion of a commercial respiratory phantom, small oscillatory motion of amplitudes lower than 0.5 mm of an in‐house phantom, and circular motion up to 11.5 cm/s. If necessary, spatial and temporal resolution could be improved by increasing the resolution and frame rate of the recording system. Using the positions of the cameras described above, the gantry and electron applicator never blocked camera views. As it was stated before, it is possible to change the position of the cameras (and perform a new calibration) if camera blockage appears.

Four hundred and sixty‐one trajectories on volunteer patients were tracked, and some conclusions about breast tumor patients motion during treatments could be done. The system could also track simultaneously 15 independent points on the thorax of a healthy volunteer. It is interesting to emphasize that in this procedure the system obtained 15 independent trajectories, so several studies could be accomplished with these data, such as average motion, angular motion of the ribcage or parts of it, and lung volume calculations. Similarly, the system would be able to obtain relative motion of parts of the body.

Based on that method, a clinical application has been developed and tested, to check patient external motion during radiation treatment and alert the operator if motion exceeds some fixed thresholds. The application has been proven useful to implement a real‐time quality control of radiotherapy treatments and actually detected some unexpected motion in several patients. As a further improvement, we could use this system to stop radiation, using the trigger of the linac, if large patient motion was detected.

## V. CONCLUSIONS

Tracking accuracy and sensitivity to high‐speed motion of the new method are high enough for patient or treatment couch tracking, similar to those of other commercial available systems. The method has other advantages. First, an arbitrary number of points can be independently tracked, all of them simultaneously, and in any region of the surface of the patient. For example, we carried out an application that needed to track 48 moving points. It makes it possible to develop comparative studies of motion of different regions of the patient, such as comparative motion of abdomen and chest. Secondly, the points to be tracked are placed on the surface of the patient, not using external pieces, so both global and partial (e.g., respiration) motion of the patient is detected with <0.3 mm uncertainty. Thirdly, the algorithm has redundant solutions, so a “double check test” is intrinsic to the system, because minor hardware errors or small motion of the measuring tools, result in discrepancies between available solutions. Finally, it has no tracking time limitation and no interference with personal or objects around the patient. Besides, the measurement setup could be easily moved and recalibrated to track points in other regions of the patient in a few minutes. Because of its simplicity of use, it could be routinely used with all the patients, with no significant delaying of the procedure.

Work is in progress to use this approach on otolaryngology and neurology problems, and in MRI and PET exams. Work is also in progress to implement an improved version of the algorithm to measure the six degrees of freedom (three of translation and three of rotation) of motion of a surface, tracking a number of points over it.

## ACKNOWLEDGMENTS

The authors acknowledge financial support of Fundación Mutua Madrileña and Spanish Government through Contract No. FIS201124642.
